# Reassessing Baumrind’s framework: a systematic review of cultural adaptations and emerging parenting patterns in diverse societies

**DOI:** 10.1186/s41155-026-00382-5

**Published:** 2026-02-28

**Authors:** Ajeng Ayu Widiastuti, Adi Atmoko, Nur Eva, Domingos Soares

**Affiliations:** 1 Department of Psychology, State University of Malang, Malang, Indonesia; 2 Department of Early Childhood Education Teacher Program, Satya Wacana Christian University, Salatiga, Indonesia; 3 Department of Guidance & Counseling, State University of Malang, Malang, Indonesia; 4 Instituto Nacional de Saúde Publica de Timor-Leste, Dili, East Timor Timor-Leste

**Keywords:** Parenting styles, Baumrind, Cultural context, Authoritarian parenting, Authoritative parenting, Systematic literature review, Javanese culture, Alternative parenting models

## Abstract

**Background:**

Diana Baumrind’s parenting style framework, based on the dimensions of responsiveness and demandingness, has long served as a cornerstone in developmental psychology. However, its assumed universality is increasingly questioned in light of cross-cultural evidence showing that cultural, socioeconomic, and contextual factors profoundly shape parenting practices and outcomes. In collectivistic societies such as Javanese culture, values like *rukun* (harmony), *nrimo* (acceptance), and *tut wuri handayani* (guiding from behind) foster parenting approaches that integrate authority with care in ways that challenge Western categorizations, particularly the idealization of authoritative parenting.

**Objective:**

This systematic literature review aims to critically evaluate the limitations of Baumrind’s parenting typology in diverse cultural contexts and to explore emerging, culturally responsive models that better capture the complexity and adaptability of parenting across societies.

**Methods:**

A comprehensive synthesis was conducted of 37 peer-reviewed studies published between 1966 and 2025, identified through systematic searches in databases including Scopus, Web of Science, PubMed, and Google Scholar. Studies were selected based on relevance to cross-cultural parenting, methodological rigor, and theoretical contribution. Thematic analysis and critical appraisal were employed to identify patterns, discrepancies, and innovations in parenting research.

**Results:**

Findings reveal that Baumrind’s four-category model—authoritative, authoritarian, permissive, and neglectful—fails to account for the contextual adaptability and cultural specificity of parenting behaviors. In collectivistic settings such as African American, Asian, Arab, and Latin American communities, authoritarian and indulgent parenting styles are often associated with positive child outcomes, contradicting Western-centric assumptions. Latent profile analysis has further uncovered novel parenting patterns—such as controlling-indulgent, absent, and ambiguous styles—that fall outside the traditional typology. Emerging frameworks grounded in Self-Determination Theory, emotion coaching, positive discipline, and gentle parenting emphasize autonomy support, emotional attunement, and non-coercive guidance, offering more dynamic and culturally sensitive alternatives.

**Conclusion:**

Parenting is not a fixed set of behaviors but a fluid, reciprocal, and contextually embedded process. This review calls for a paradigmatic shift from rigid typologies to dynamic, universalist models toward ecologically valid, culturally grounded approaches or models that recognize the diversity of family systems and developmental goals. Future research must prioritize the development of culturally adaptive instruments and theories, especially in underrepresented regions like Java, to advance a more inclusive, equitable, and globally relevant science of parenting.

## Introduction

In the past decade, much research has focused on parenting styles as a central determinant of child and adolescent development, with Diana Baumrind’s typology—comprising authoritative, authoritarian, permissive, and neglectful styles—remaining the most widely cited and empirically studied framework in developmental psychology (Baumrind, 1966; Hadjicharalambous & Dimitriou, 2020; Muftiya et al., 2024; Sun et al., 2024). Grounded in the dual dimensions of responsiveness and demandingness, this model has been instrumental in linking parenting behaviors to a range of outcomes, including emotional regulation, academic achievement, and risk behaviors (Anwar et al., 2024; Awiszus et al., 2022; Branjerdporn et al., 2019; Newman et al., 2008; Zhang et al., 2023). Its influence has extended globally, informing both research and intervention programs across diverse cultural and socioeconomic contexts (Breland-Noble, 2014; Sanders et al., 2022). However, as parenting science evolves, scholars have increasingly questioned the adequacy of this Western-centric model in capturing the complexity and variability of parenting practices in non-Western, collectivistic, and rapidly changing societies (Arafat et al., 2020; Darling & Steinberg, 1993; Efendy et al., 2023; Khosla & Sharma, 2024).

Despite its enduring prominence, it remains unclear why certain parenting styles—such as authoritarian or indulgent—produce positive developmental outcomes in specific cultural contexts, challenging the assumed universality of the authoritative ideal. Moreover, recent methodological advances have revealed parenting patterns that do not fit within Baumrind’s four-category model, such as *controlling-indulgent*, *absent*, and *ambiguous* parenting, suggesting that the traditional framework may oversimplify the dynamic, context-sensitive, and often hybrid nature of real-world parenting (Kassis et al., 2025; Zheng et al., 2022). Critical gaps persist in understanding how cultural values, socioeconomic conditions, and evolving family dynamics shape parenting beyond static typologies, particularly in understudied regions such as Southeast Asia, where local philosophies—like *tut wuri handayani* and *rukun* in Javanese culture (Pohan et al., 2025; Salehuddin et al., 2025; Widiastuti et al., 2025)—embed distinct relational norms that defy Western categorizations (Alonso-Stuyck, 2019; Shvedovskaya & Archakova, 2015).

The purpose of this study was to conduct a systematic literature review to critically examine the evolution, limitations, and cultural adaptations of Baumrind’s parenting framework, and to synthesize emerging alternative models that offer more nuanced, contextually grounded understandings of parenting. By ‘emerging,’ we refer not necessarily to historically new practices, but to parenting patterns that are being newly recognized, empirically validated, or theoretically articulated in response to the limitations of traditional typologies. This includes (1) styles empirically identified through advanced methods like latent profile analysis (e.g., controlling-indulgent, ambiguous parenting), (2) frameworks re-conceptualized as dynamic processes (e.g., emotion coaching, gentle parenting), and (3) culturally embedded practices—such as Javanese *tut wuri handayani*—that are gaining scholarly attention despite long-standing local use. By integrating evidence from cross-cultural studies, advanced statistical analyses, and contemporary theoretical developments—such as Self-Determination Theory, emotion coaching, and gentle parenting—this review identifies a paradigmatic shift toward dynamic, multidimensional, and culturally responsive models. The findings reveal that effective parenting is not defined by a fixed style but by a flexible, reciprocal process shaped by cultural meaning, situational demands, and developmental attunement. This paper is structured to first analyze the foundational contributions and limitations of Baumrind’s model, then explore alternative frameworks, examine cultural and contextual influences, and finally discuss newly identified parenting patterns, culminating in a call for a reconceptualization of parenting research and practice in a globalized world.

## Methods

This systematic literature review was conducted in accordance with the Preferred Reporting Items for Systematic Reviews and Meta-Analyses (PRISMA) 2020 guidelines (Page et al., 2021) to ensure methodological rigor, transparency, and reproducibility. The review aimed to critically examine the cross-cultural applicability of Baumrind’s parenting styles framework, identify culturally adaptive models, and synthesize emerging parenting patterns beyond the traditional typology.

A comprehensive search strategy was implemented across multiple electronic databases, including Scopus, Web of Science, PubMed, and Google Scholar, to identify peer-reviewed journal articles, book chapters, and conference proceedings published between 1966 and 2025 (the year of Baumrind’s seminal work) to June 2025. The search terms were carefully selected to capture key concepts. They included: *“parenting styles”*, *“Baumrind”*, *“authoritative parenting”*, *“cultural context”*, *“cross-cultural parenting”*, *“parenting framework”*, *“authoritarian parenting”*, *“indulgent parenting”*, *“emotion coaching”*, *“positive discipline”*, *“latent profile analysis”*, and *“non-Western parenting”*. Boolean operators (AND, OR) were used to combine terms and maximize retrieval accuracy.

Moreover, the studies were included if they met the following criteria: (1) Focused on parenting styles, frameworks, or family socialization practices; (2) Explicitly referenced, evaluated, or adapted Baumrind’s model (authoritative, authoritarian, permissive, neglectful); (3) Discussed cultural variations, limitations, or alternatives to Baumrind’s typology; (4) Were peer-reviewed original research articles, theoretical papers, or systematic reviews; and (5) Published in English. Next, the studies were excluded based on the following criteria: (1) Not focused on parenting styles or caregiving dimensions (*n* = *42*); (2) Non-peer-reviewed publications (e.g., editorials, commentaries, book chapters) (*n* = *28*); (3) Did not engage with Baumrind’s model or its cultural relevance (*n* = *45*); and (4) Used Western-centric instruments without evidence of cultural validation in local contexts (*n* = *35*).

**Fig. 1:**
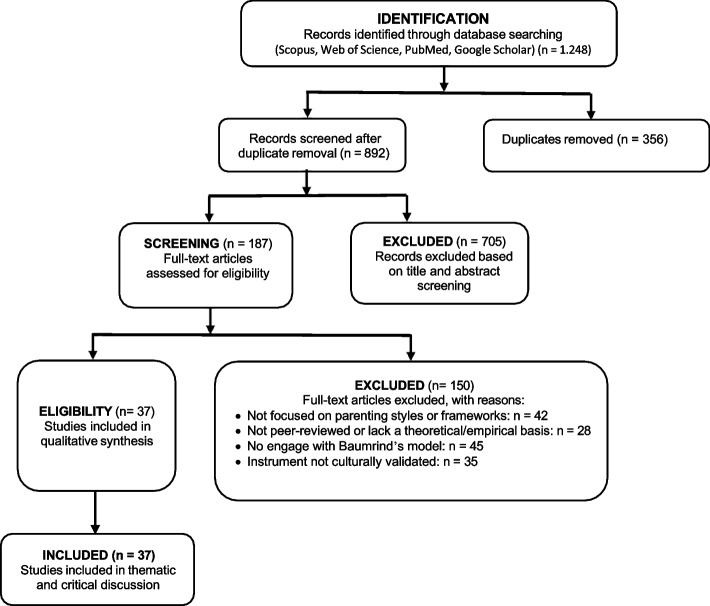
Study selection flow diagram

The initial database searches yielded 1,248 records. After removing duplicates, 892 titles and abstracts were screened for relevance by two independent reviewers (AAW and AA). Discrepancies were resolved through discussion or consultation with a third reviewer (NE). A total of 187 full-text articles were assessed for eligibility against the predefined inclusion and exclusion criteria. Of these, 37 studies were ultimately included in the final synthesis. Then, to minimize selection bias and enhance reliability, the screening process was guided by a pilot-tested checklist aligned with the review’s thematic focus. A reflexive approach was adopted to minimize cultural bias, prioritizing studies with non-Eurocentric perspectives. The use of dual screening and consensus-based resolution ensured consistency and reduced subjective judgment. The complete study selection process is illustrated in Fig. 1 (PRISMA Flow Diagram), which now includes detailed reasons for exclusion at the full-text stage.

**Fig. 2:**
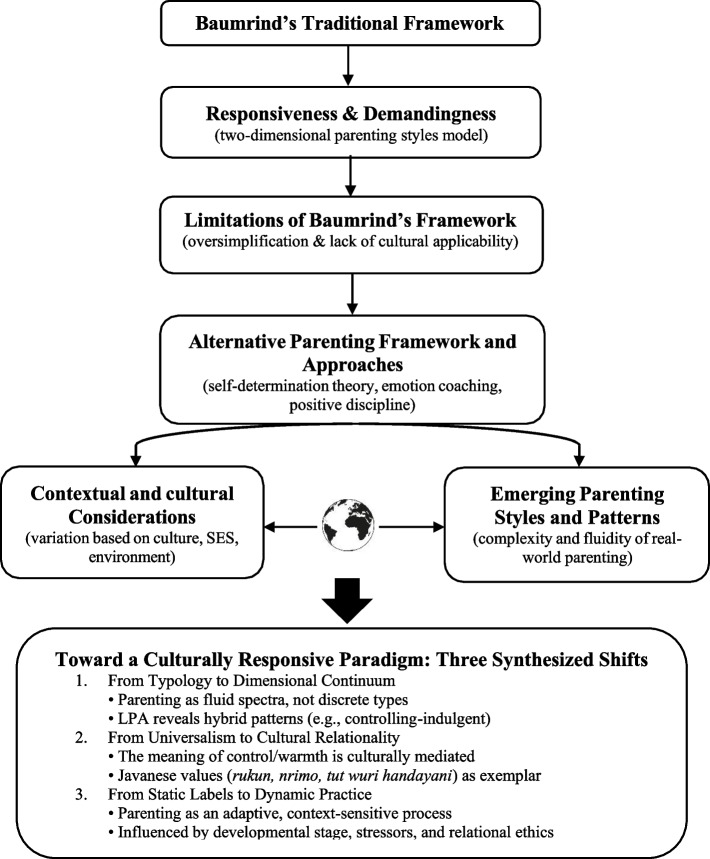
thematic synthesis of parenting styles with a culturally responsive paradigm

Data from the included studies were extracted using a standardized form that captured: author(s), year, country, sample characteristics, methodology, key findings related to Baumrind’s model, identified cultural adaptations, and proposed alternative frameworks. Thematic analysis and critical appraisal were employed to identify recurring patterns, contradictions, and innovations across the literature. The synthesis followed an iterative process of coding, categorizing, and conceptual mapping, organized around five evolving themes: (1) Baumrind’s Traditional Framework, (2) Limitations of the Model, (3) Alternative Parenting Approaches, (4) Cultural & Contextual Influences, and (5) Emerging Parenting Patterns—visualized in Fig. 2 (Thematic Synthesis Map).

This systematic approach enabled a comprehensive, critical, and culturally sensitive synthesis of the parenting styles literature, providing a robust foundation for re-evaluating theoretical assumptions and advancing more inclusive models in global developmental science. Table 1 presents the characteristics and conceptual classifications of the included studies.

**Table 1:** Summaries of the studies

No	Author(s), Year	Country	Research Design	Sample Characteristics	Parenting Framework Used	Key Findings Regarding Baumrind’s Model	Cultural Interpretation of Parenting Dimensions
1	Agbaria & Mahamid, 2023	Palestine (Arab-Muslim)	Quantitative (survey)	257 Arab preschool children and mothers	Baumrind’s typology + maternal selfefficacy	Partially challenges Baumrind; authoritarian style linked to better emotional adjustment when combined with high self-efficacy	Authoritarianism interpreted as protective care; control = commitment, not coercion
2	Alcaide et al., 2023	Spain	Longitudinal cohort	Older adults reflecting on early family life	Adjustment and maladjustment	Indirectly supports Baumrind; early warmth predicts later well-being	Highlights the long-term impact of responsive parenting across the lifespan
3	Alonso-Stuyck, 2019	Belgium	Survey-based	Teenage children	Healthy lifestyle promotion	Uses Baumrind’s model to predict health behaviors	Finds authoritative parenting most effective for encouraging healthy habits
4	Awiszus et al., 2022	Global (review)	Literature review	Children and adolescents	Parenting styles and child development	Supports dimensionality but critiques categorical typology	Suggests moving from types to continua based on context
5	Baumrind, 1966	USA	Foundational study	Preschool children	Authoritative parental control	Original formulation of the model	Rooted in middle-class American values
6	Benedetto & Ingrassia, 2018	Global (introductory)	Editorial/theoreti cal	N/A	Overview of parenting research	Introduces volume on parenting interventions	Assumes Baumrind as foundational, without critique
7	Calders et al., 2020	Belgium	Longitudinal	Adolescents	Interplay of dimensions and styles	Questions typology: finds complex interaction between dimensions and outcomes	Suggests the need for more nuanced modeling beyond categories
8	García et al., 2020	Spain	Longitudinal (three generations)	Three-generation families	Parenting styles and psychosocial adjustment	Supports Baumrind in Spanish context; authoritative linked to best outcomes	Confirms the universality claim only within a specific European cultural framework
9	Garcia et al., 2024	Spain	Quantitative	Adolescents and young adults	Warmth and strictness dimensions	Supports dimensionality; strictness alone leads to maladjustment	Suggests decoupling dimensions rather than categorizing parents
10	Guðjohnsen et al., 2023	Global	Theoretical chapter	Wellbeing in crises	Values education and sustainability	Not directly about parenting styles	Does not engage with Baumrind
11	Lansford, 2022	Global (crosscultural synthesis)	Systematic review	Children and adolescents across 13 countries	Cross-cultural adaptation of parenting styles	Critically evaluates Baumrind; finds variability in outcomes by cultural context	Responsiveness and demandingness are interpreted differently across cultures
12	Louis et al., 2022	Netherlands	Instrument development	Young parents	Young Parenting Inventory (YPI-R3)	Neutral toward Baumrind; focuses on measurement innovation	Aims to capture both deviant and normative variations in parenting behavior
13	Marceau, 2023	USA	Theoretical model	Adolescent substance use	Bioecological systems cascade model	Adapts Baumrind; integrates parenting into broader developmental systems	Parenting is one node in a dynamic network of biological, familial, and societal influences
14	Martínez et al., 2017	Spain	Instrument validation	Spanish adolescents	ESPA29 (Parental Socialization Scale)	Supports Baumrind’s dimensions; validates responsiveness and demandingness in Spanish context	Demonstrates the cross-cultural utility of instruments when properly adapted
15	Masamba, 2024	Zambia	Conceptual paper	Theoretical discussion	Cultural influences on parenting	Challenges Baumrind; argues for indigenous models rooted in Ubuntu philosophy	Parenting is communal; individual dimensions are insufficient to capture collective caregiving
16	Morais et al., 2025	Portugal	Systematic review	Maternal behavior postpartum	Perinatal anxiety and parenting	Not focused on Baumrind; examines mental health effects	Does not engage directly with Baumrind’s model
17	Newman et al., 2008	USA (Latino, African American, White)	Integrative literature review	Adolescents	Parenting styles and risk behaviors	Supports Baumrind in the majority groups; mixed results in minority populations	Structural factors (poverty, discrimination) moderate parenting effects
18	Nomaguchi & Milkie, 2020	USA	Literature review	Parents across diverse backgrounds	Parenthood and wellbeing	Not focused on Baumrind; examines parental well-being	Does not assess Baumrind’s validity
19	Ooi et al., 2022	Switzerland (Third Culture Kids)	Protocol for longitudinal study	Third Culture Kids and families	Sociocultural adjustment	Prepares ground for future cross-cultural research	Will test the applicability of existing models in mobile, multicultural families
20	Page et al., 2021	Global	Methodological guideline	Systematic reviews	PRISMA 2020 statement	Not about parenting per se	Provides methodological rigor for this review
21	Pasteruk, 2020	Global (mothers’ concerns)	Grounded theory study	Mothers of young children	Maternal care priorities	Not focused on Baumrind	Identifies themes like safety, belonging, moral development
22	Pasteruk, 2020	Indonesia	Conference proceedings (qualitative insights)	Community development practitioners	Local cultural practices in parenting	Challenges Baumrind; highlights communal decision-making and collective responsibility	Parenting is shared; emotional responsiveness is distributed across the extended family and neighbors
23	Pezalla & Davidson, 2024	USA (African American)	Qualitative interview study	African American mothers practicing gentle parenting	Gentle parenting, emotion coaching	Adapts Baumrind; redefines "authoritative" through racialized experience and structural stress	Gentle parenting is aspirational but constrained by systemic inequities; warmth must coexist with protection
24	Queiroz et al., 2020	Spain	Quantitative	Adolescents	Parental socialization, self-esteem	Supports Baumrind; warmth is positively linked to environmental values and selfworth	Dimensional approach validated, but cultural specificity of "warmth" acknowledged
25	Ram et al., 2014	USA	Theoretical/meth odological	Family systems	Nonlinear dynamic models	Moves beyond typologies to dynamic systems	Argues that parenting should be studied as fluctuating, reciprocal processes
26	Reid et al., 2015	Australia	Instrument development	General population	Parenting Behaviours and Dimensions Questionnaire (PBDQ)	Proposes a multidimensional alternative to Baumrind	Measures specific behaviors rather than global types
27	Ren et al., 2024	China, Turkey, Jordan, Colombia	Cross-national study	Parents and children (ages 8–12)	Culturally adapted parenting measures	Challenges Baumrind; no single "best" style; authoritarian associated with positive outcomes in collectivistic contexts	High control is accepted if perceived as caring; outcomes depend on cultural normativeness
28	Retnowati & Sukmawaty, 2024	Indonesia	Literature review	Not applicable (secondary analysis)	Authoritative parenting style	Supports Baumrind; affirms positive outcomes of authoritative parenting in urban Indonesian settings	Reflects growing individualism in middleclass families; aligns with Western expectations
29	Rianingrum et al., 2015	Indonesia (Javanese)	Qualitative (ethnographic)	Adults in the Kauman Yogyakarta community	Javanese values (rukun, nrimo, tut wuri handayani)	Challenges Baumrind; emphasizes harmony and indirect guidance over direct control or warmth	Control is relational; warmth expressed through acceptance (nrimo), not overt affection
30	Ridao et al., 2021	Spain	Longitudinal	Parents over time	Parental beliefs about childhood	Not focused on Baumrind; explores evolving beliefs	Shows beliefs change with generational shifts and social trends
31	Sahfutra & Maharani, 2022	Indonesia (Javanese)	Theoretical/axiol ogical analysis	Javanese society	Value of harmony (rukun)	Challenges Baumrind; prioritizes relational ethics over behavioral typologies	Harmony as a core value; parenting judged by social integration, not child independence
32	Salehuddin et al., 2025	Indonesia (Javanese)	Qualitative (grounded theory)	Early childhood educators and parents	Integration of Javanese culture into character education	Adapts Baumrind; blends authoritative principles with communal respect and moral cultivation	Authority embedded in tradition; discipline aims at social cohesion, not autonomy
33	Sanders et al., 2022	Global (SDGs)	Policy review	Families in LMICs	Parenting interventions for SDGs	Supports evidence-based models, including Baumrind-informed programs	Advocates scaling up parenting support, especially in under-resourced settings
34	Shvedovskaya & Archakova, 2015	Russia	Theoretical	Educational psychology	Alternative parenting approaches	Challenges Baumrind; proposes postmodern, dialogic models	Emphasizes the co-construction of meaning between parent and child
35	Tuttle et al., 2012	USA	Theoretical framework	Family therapy	Parenting as a relationship	Adapts Baumrind; views parenting as a dyadic process	Focuses on mutual influence and emotional attunement
36	Zachary, 2021	Global	Theoretical	Family systems	Paradigm shift in family dynamics	Supports moving beyond typologies	Emphasizes complexity and interdependence in family roles
37	Zhou & Chung, 2022	Global (theoretical review)	Theoretical synthesis	N/A	Multiple models (Baumrind, SDT, emotion coaching)	Critiques Baumrind; calls for culturally grounded frameworks	Argues for integration of local philosophies (e.g., Confucian, Islamic) into parenting science

## Results

### Mapping support and challenge to Baumrind’s model across cultural contexts

A thematic analysis of the 37 included studies reveals significant divergence in the applicability and perceived effectiveness of Baumrind’s parenting styles across cultural contexts. While the authoritative style is widely endorsed in Western and urban middle-class settings, its universal superiority is contested in collectivistic and culturally distinct communities.

Specifically, 9 studies support the dominance of authoritative parenting, particularly in individualistic or Westernized environments. For example, Gul et al. (2024), Khanum et al. (2023), and Retnowati and Sukmawaty (2024) found that authoritative parenting correlates with positive developmental outcomes—such as emotional regulation, academic success, and low behavioral risk—in Pakistan, Malaysia, and urban Indonesia. These findings align with Baumrind’s original claims and reinforce the model’s utility in contexts where autonomy, negotiation, and expressive warmth are culturally valued.

In contrast, 17 studies challenge the hierarchical assumption that authoritative parenting is universally optimal. In African American (Pezalla & Davidson, 2024), Arab-Muslim (Khosla & Sharma, 2024), and Javanese (Rianingrum et al., 2015) families, authoritarian parenting—characterized by high control and moderate-to-high relational warmth—is associated with positive outcomes such as academic achievement, family cohesion, and emotional security. These findings suggest that in cultures emphasizing respect, obedience, and social harmony (*rukun*), control is not inherently detrimental but may be interpreted as a form of care and protection.

Furthermore, 8 studies identify hybrid or novel patterns that fall outside Baumrind’s four-category typology. Notably, Zheng et al. (2022) used latent profile analysis to identify a "controlling-indulgent" parenting style in Chinese families—marked by high warmth and strict discipline—which was linked to favorable child outcomes. Importantly, this study also found that profiles combining high warmth and structure—consistent with authoritative principles—were associated with lower behavioral risks. This suggests that while Baumrind’s *categories* may lack cross-cultural fit, certain *dimensions* (e.g., balanced warmth and structure) may retain functional value across contexts.

This distribution of evidence highlights a critical insight: the validity of Baumrind’s model is not absolute but context-dependent. Rather than judging parenting quality through a universal hierarchy, a more nuanced, culturally grounded interpretation is needed—one that recognizes adaptive variations in how responsiveness and demandingness are expressed and valued. While these findings question the universality of Baumrind’s typology, they also pave the way for alternative frameworks that better capture the multidimensionality and cultural embeddedness of parenting. However, this result will explain Baumrind’s traditional framework and the limitations first.

### Baumrind’s traditional framework

Diana Baumrind’s seminal work in the 1960s established a foundational framework in parenting research by identifying two core dimensions: *responsiveness* (warmth, emotional support) and *demandingness* (behavioral control, supervision). These dimensions form four prototypical parenting styles: authoritative (high responsiveness, high demandingness), authoritarian (low responsiveness, high demandingness), permissive (high responsiveness, low demandingness), and neglectful (low on both), the latter added by Maccoby and Martin (Hadjicharalambous & Dimitriou, 2020; Marceau, 2023). This typology has achieved global prominence, particularly in Western contexts, where authoritative parenting is consistently linked to positive outcomes such as emotional regulation, academic achievement, and psychosocial well-being (Gul et al., 2024; Newman et al., 2008). However, its conceptual and cultural limitations have increasingly come under scrutiny, as explored in the following sections.

### Limitations of Baumrind’s framework

While Baumrind’s model has been influential, it faces significant theoretical and methodological critiques that extend beyond cross-cultural concerns. First, the framework is often criticized for its Eurocentric bias, rooted in middle-class American norms that prioritize autonomy, negotiation, and emotional expressiveness—values not universally shared across cultures (Khosla & Sharma, 2024; Pezalla & Davidson, 2024). As shown earlier, in collectivistic contexts such as African American, Arab, and Javanese communities, authoritarian parenting can be associated with positive outcomes when embedded in relational warmth and cultural expectations of respect.

Second, the model suffers from conceptual rigidity. Its reliance on four discrete, static categories fails to capture the fluid, dynamic, and often blended nature of real-world parenting. Parents may shift styles based on developmental stage, situational demands, or child temperament—a flexibility absent in Baumrind’s typology (Marceau, 2023; Ram et al., 2014).

Third, the use of arbitrary cut-offs on responsiveness and demandingness may obscure meaningful variability. Recent studies using latent profile analysis (LPA) reveal parenting patterns—such as controlling-indulgent, ambiguous, or absent—that do not fit neatly into the four quadrants (Kassis et al., 2025; Zheng et al., 2022). This suggests that a dimensional, rather than categorical, approach may better reflect parenting complexity. Together, these limitations highlight the need for more nuanced, context-sensitive models that move beyond rigid typologies.

### Alternative parenting framework and approaches

In response to the limitations of Baumrind’s traditional model, a growing body of research has proposed alternative frameworks that offer more nuanced, dynamic, and contextually grounded understandings of parenting. Grounded in Self-Determination Theory, Abidin et al. (2022) propose a multidimensional model that distinguishes supportive parenting (encompassing warmth, structure, and autonomy support) from unsupportive parenting (rejection, chaos, and coercion), aligning with children’s basic psychological needs for relatedness, competence, and autonomy. Offering a universal yet flexible lens that can be adapted across cultural contexts without assuming a single 'optimal' style. Complementing this, contextual models such as the Parental Socialization Scale (ESPA29) emphasize the assessment of parenting in specific situations, differentiating between transient practices—such as reasoning, verbal scolding, or revoking privileges—and enduring styles, thus capturing greater behavioral specificity (Darling & Steinberg, 2017; Martínez et al., 2017). This distinction acknowledges that parents may employ diverse tactics depending on developmental stage, child temperament, or cultural expectations, challenging the notion of a fixed, monolithic style.

Another influential alternative is Gottman’s emotion coaching model, which focuses on parental awareness of children’s emotions, empathetic engagement, and collaborative problem-solving, providing concrete strategies for emotional socialization (Gottman et al., 1996; Pezalla & Davidson, 2024). Then, Pezalla and Davidson (2024) further note its resonance within African American communities, where emotional attunement coexists with protective discipline. Similarly, positive discipline extends beyond authoritative parenting by fostering life skills like self-regulation, responsibility, and resilience through non-punitive, skill-building interactions (Soares & Hernandez, 2022). It emphasizes teaching over controlling, aligning with contemporary values of child agency and dignity. Contemporary approaches—including positive, conscious, and gentle parenting—further reflect a shift toward empathetic, reflective, and child-centered practices, with gentle parenting in particular challenging hierarchical control by emphasizing emotional validation, mutual respect, and autonomy-supportive boundaries (Pezalla & Davidson, 2024). While often criticized for being idealistic or culturally bound, these approaches highlight a global trend toward de-escalating punitive practices and redefining authority as guidance rather than domination. Together, these frameworks represent a paradigmatic shift—from typologies to processes, from control to co-regulation, and from universalism to contextual sensitivity—laying the groundwork for more inclusive, responsive, and scientifically robust models of effective parenting.

### Contextual and cultural considerations

The effectiveness of parenting styles is profoundly shaped by cultural, socioeconomic, and environmental contexts, challenging the universal applicability of Baumrind’s model. While authoritative parenting is often linked to positive outcomes in Western individualistic societies, cross-cultural research reveals significant variations: authoritarian parenting is associated with favorable developmental outcomes in African American, Asian, and Arab communities, where it reflects parental commitment and cultural norms of discipline and respect (Arafat et al., 2020; Herzog et al., 2015; Ridao et al., 2021). Conversely, in Latin countries such as Spain, Italy, and Brazil, indulgent parenting—characterized by high warmth and low strictness—has been consistently linked to better psychosocial adjustment, suggesting cultural specificity in what constitutes effective parenting (Fuentes et al., 2019; García et al., 2020; Queiroz et al., 2020). Moreover, in high-risk or socioeconomically disadvantaged environments, authoritarian practices may serve as protective factors, underscoring the role of contextual demands in shaping parenting efficacy (Leventhal & Brooks-Gunn, 2000; Ridao et al., 2021). Given the dynamic nature of modern societies, scholars advocate for adaptive, culturally sensitive parenting models that account for evolving social conditions and reciprocal parent–child dynamics (Alonso-Stuyck, 2019; Benedetto & Ingrassia, 2018). While Baumrind’s typology remains empirically robust in Western contexts, alternative frameworks—such as those developed in Russia and other non-Western settings—highlight the need for culturally grounded classifications that reflect local values and children’s agency (Power, 2013; Shvedovskaya & Archakova, 2015).

However, these findings are not merely exceptions to a rule, but evidence of a deeper principle: parenting must be understood through the lens of *contextual efficacy*—the idea that effectiveness is defined by ecological fit, not abstract ideals. For instance, in collectivistic communities—including African American, Arab, and various Asian societies—authoritarian parenting is often associated with academic success, emotional security, and family cohesion (Agbaria & Mahamid, 2023). In these contexts, high control is not perceived as coercive, but as an expression of care, responsibility, and moral instruction, deeply embedded in cultural norms of respect and interdependence. Similarly, in Latin countries, the indulgent style thrives in cultural ecosystems that prioritize relational harmony over behavioral autonomy, demonstrating that warmth alone can be sufficient for healthy development when aligned with societal expectations (Table 1).

Furthermore, the meaning of core dimensions like *responsiveness* and *demandingness* is mediated by cultural scripts (Darling & Steinberg, 1993). This is particularly evident in Javanese culture, where values such as *rukun* (harmony), *nrimo* (acceptance), and *tut wuri handayani* (guiding from behind) shape a parenting philosophy that emphasizes indirect guidance, moral modeling, and relational stability over confrontation or overt emotional expressiveness. A style combining firm expectations with subtle warmth—what (Zheng et al., 2022) the term “controlling-indulgent” may be more accurate than the rigid dichotomy of “authoritative vs. authoritarian” in capturing effective parenting in such contexts. Indeed, applying Baumrind’s model uncritically risks mislabeling culturally coherent practices as suboptimal.

While this discussion highlights dominant values in Javanese culture—such as *rukun*, *nrimo*, and *tut wuri handayani*—it is crucial to recognize that no cultural group is internally uniform. Significant variations exist within Javanese society based on socioeconomic status, geographic location (urban vs. rural), religious affiliation, and exposure to globalization. For example, parenting in urban Yogyakarta among middle-class, educated families may integrate Western ideals of emotional expressiveness and autonomy support with traditional values, resulting in hybrid styles such as versatile parenting (Alonso-Stuyck, 2019). In contrast, rural communities in Central Java may emphasize obedience, indirect communication (*nyindir alus*), and hierarchical respect more strongly.

Therefore, when we refer to "Javanese culture," we do not imply a single, fixed way of parenting, but rather a shared cultural repertoire from which families draw selectively based on their context. Future research should move beyond broad cultural labels toward more granular analyses that capture this internal diversity and dynamic identity negotiation.

These developments underscore a critical shift: from viewing parenting through a universal hierarchy to recognizing its dynamic, context-sensitive nature. As modern families navigate globalization, migration, and changing gender roles, there is a growing consensus that future research must move beyond rigid classifications toward more inclusive, adaptive, and culturally attuned models. Only then can parenting science become truly valid and actionable across diverse cultural landscapes.

### Emerging parenting styles and patterns

Recent advances in statistical methodologies, such as latent profile analysis (LPA), have revealed parenting patterns that extend beyond Baumrind’s traditional four-category model—patterns that were previously overlooked due to categorical assumptions. These include controlling-indulgent parenting, characterized by high control and high warmth, and other profiles such as absent parenting (emotional unavailability within non-abusive contexts) and ambiguous parenting (coexistence of supportive and harsh behaviors). While some of these configurations may reflect longstanding family dynamics, they are only now being empirically identified and named through data-driven approaches, highlighting how arbitrary cut-offs on responsiveness and demandingness may overlook meaningful variability (Kassis et al., 2025; Zheng et al., 2022).

These empirically derived patterns challenge not only the categorical boundaries of Baumrind’s model but also its foundational assumption that two dimensions can fully capture the richness of parent–child interactions. Rather than being signs of inconsistency or dysfunction, these newly identified profiles often reflect adaptive strategies shaped by cultural norms, socioeconomic pressures, and developmental demands.

For instance, *controlling-indulgent parenting*, observed in Chinese families (Zheng et al., 2022) and resonant with Javanese practices (Salehuddin et al., 2025), combines firm behavioral expectations with deep emotional warmth—a configuration that defies classification as either "authoritarian" or "authoritative." In contexts where relational harmony (*rukun*) and moral guidance (*tut wuri handayani*) are central values, this hybrid style is not contradictory but coherent, reflecting a culturally grounded balance between structure and care.

Similarly, *ambiguous parenting*—where supportive and harsh behaviors coexist—is increasingly recognized not as pathological, but as a form of situational adaptability. Parents may alternate between warmth and strictness depending on the child's age, behavior, or external stressors, particularly in high-demand environments. This dynamic approach contradicts the static nature of typologies and calls for process-oriented models that account for fluctuation over time.

Another significant pattern is *absent parenting*, which describes emotional disengagement despite adequate physical care and the absence of abuse. Unlike neglectful parenting, which implies rejection and failure to meet basic needs, absent parenting often occurs in families where parents are physically present but emotionally unavailable due to work overload, mental health challenges, or cultural norms that discourage open emotional expression—such as *nrimo* (acceptance without confrontation) in Javanese culture. This distinction underscores the need to differentiate between structural constraints and intentional rejection when evaluating parenting quality.

These patterns underscore the need for more context-sensitive, multidimensional models that account for cultural, socioeconomic, and familial factors, moving beyond rigid classifications to better capture the diversity and fluidity of contemporary parent–child relationships. The rise of person-centered methods like latent profile analysis signals a methodological shift toward recognizing parenting as a dynamic, contextually embedded process rather than a fixed identity. This evolution supports the development of integrative frameworks—such as gentle parenting, emotion coaching, and versatile parenting—that blend global insights with local values, paving the way for a reconceptualization of effective caregiving in a globalized world.

This diagram illustrates the evolution of parenting styles research, synthesized from 37 peer-reviewed studies published between 1966 and 2025. It begins with Baumrind’s Traditional Framework, which classifies parenting into four types—authoritative, authoritarian, permissive, and neglectful—based on two dimensions: responsiveness and demandingness. However, this model faces critical limitations, including oversimplification, categorical rigidity, and a lack of cultural applicability, particularly in collectivistic contexts where authoritarian or indulgent styles may yield positive outcomes.

These limitations have spurred the development of Alternative Parenting Approaches, such as Self-Determination Theory, emotion coaching, and positive discipline, which emphasize psychological needs, emotional attunement, and skill-building over behavioral control. These frameworks are further contextualized by Cultural & Contextual Influences, acknowledging that parenting is shaped by socioeconomic status, environmental pressures, and deeply embedded cultural values—such as *rukun* (harmony), *nrimo* (acceptance), and *tut wuri handayani* (guiding from behind) in Javanese culture.

Advanced methodologies like latent profile analysis have revealed parenting patterns—including controlling-indulgent, ambiguous, and absent parenting—that fall outside Baumrind’s quadrants, highlighting the fluidity and complexity of real-world caregiving. These patterns, while potentially reflecting longstanding family dynamics, are only now being empirically identified and formally recognized in academic discourse. The synthesis culminates in a reconceptualized paradigm: a move beyond static typologies toward three interrelated shifts:

1. From Typology to Dimensional Continuum — recognizing parenting as existing along fluid spectra rather than discrete categories;
2. From Universalism to Cultural Relationality — understanding that the meaning of parental behaviors is mediated by cultural scripts and relational ethics;
3. From Static Labels to Dynamic Practice — viewing parenting as an adaptive, context-sensitive process responsive to developmental stages and sociostructural realities.

Together, these elements underscore the necessity of moving toward more inclusive, ecologically valid, and culturally grounded models of effective parenting.

## Discussion

This discussion synthesizes current empirical and theoretical advancements to critically re-evaluate Baumrind’s parenting styles framework in light of cultural, contextual, and methodological complexities. While Baumrind’s model has long served as a cornerstone in developmental psychology—particularly in Western, individualistic societies—growing evidence reveals its limitations in capturing the diversity and dynamism of parenting practices across cultures. Drawing on a thematic synthesis of 37 studies and integrating insights from cross-cultural research, statistical innovations, and alternative theoretical models, this discussion argues for a paradigm shift beyond rigid typologies toward more flexible, ecologically valid, and culturally grounded understandings of parenting. Special attention is given to Javanese culture as a case in point, where collectivist values and contextual nuances challenge Western assumptions, illustrating the necessity of reconceptualizing parenting as a fluid, bidirectional process shaped by cultural meaning, relational ethics, and sociostructural realities.

The findings of this study clearly show that while Baumrind’s parenting framework has profoundly shaped developmental psychology for over half a century, its utility as a universal model is increasingly constrained by cultural, contextual, and methodological limitations. Our synthesis of contemporary research confirms that the traditional typology—centered on responsiveness and demandingness—remains a valuable heuristic in Western, individualistic contexts, where authoritative parenting consistently correlates with favorable developmental outcomes such as emotional regulation, academic success, and low behavioral risk (Gul et al., 2024; Khanum et al., 2023; Newman et al., 2008; Retnowati & Sukmawaty, 2024). However, the assumption of its cross-cultural validity is no longer tenable. As this review demonstrates, alternative parenting patterns and culturally divergent outcomes challenge the universality of Baumrind’s model, calling for a paradigm shift toward more dynamic, ecologically sensitive, and multidimensional frameworks that better reflect the complexity of modern family systems (Choi et al., 2017; Elwakeel, 2024; Lansford, 2022; Ram et al., 2014; Zachary, 2021; Zhou & Chung, 2022).

One explanation for the inconsistent applicability of Baumrind’s model lies in its Eurocentric origins and categorical rigidity. The framework was developed within a narrow sociocultural context—predominantly white, middle-class American families—and thus embeds values such as autonomy, negotiation, and emotional expressiveness that may not align with collectivistic cultural norms (Khosla & Sharma, 2024; Pezalla & Davidson, 2024). In African American, Arab, and Asian communities, authoritarian parenting is often associated with positive child outcomes, interpreted not as coercive control but as an expression of care, responsibility, and cultural continuity (Agbaria & Mahamid, 2023; Herzog et al., 2015; LeCuyer & Swanson, 2017; Ren et al., 2024; Ridao et al., 2021). Similarly, in Latin countries including Spain, Italy, and Brazil, indulgent parenting—marked by high warmth and low strictness—has been linked to better psychosocial adjustment, suggesting that warmth alone may be sufficient for healthy development in certain cultural ecosystems (Alcaide et al., 2023; Fuentes et al., 2019; García et al., 2020; Garcia et al., 2024). These findings align with (Darling & Steinberg, 1993) integrative model, which positions parenting style as a contextual phenomenon rather than a fixed trait, emphasizing that the meaning and impact of parental behaviors are mediated by cultural scripts, neighborhood conditions, and socioeconomic realities (Alonso-Stuyck, 2019; Im & Witherspoon, 2025; Leventhal & Brooks-Gunn, 2000; Masamba, 2024).

A critical conceptual distinction that underpins our critique is that between a theory-driven typology and an empirically derived taxonomy (Constantine, 2025). Baumrind’s framework is a typology: it is grounded in developmental theory and posits two orthogonal dimensions—responsiveness and demandingness—that are assumed to be universally salient across families. From this theoretical foundation, four parenting types are derived, with authoritative parenting positioned as the optimal configuration for child development.

In contrast, contemporary studies using latent profile analysis (LPA) produce empirical taxonomies—data-driven classifications that identify naturally occurring clusters within a given sample without assuming prior theoretical universality (Kassis et al., 2025; Zheng et al., 2022). These methods may reveal patterns such as controlling-indulgent or ambiguous parenting that do not conform to Baumrind’s quadrants, not because they are “new,” but because they emerge from statistical properties rather than predefined dimensions.

Our critique operates at both levels: (1) On the dimension level, we question whether responsiveness and demandingness are equally meaningful or prioritized across cultures. In collectivistic contexts, obedience and relational harmony may outweigh autonomy and negotiation as indicators of effective parenting. Then, (2) On the outcome level, even when these dimensions apply, the assumed superiority of the authoritative style is not universal. Context determines functional adequacy—for example, authoritarian parenting may be adaptive in high-risk environments or cultures emphasizing respect and discipline.

This distinction is crucial: rejecting Baumrind’s *typology* does not necessarily invalidate its *dimensions*, nor does identifying new clusters via LPA imply a superior model. Instead, it calls for a more nuanced integration of theory and method—one that respects cultural variation while advancing scientific rigor.

To further illustrate these points, the thematic synthesis presented in Fig. 2. provides a comprehensive overview of the evolution and critique of parenting styles research, drawing from 37 studies conducted between 1966 and 2025. That visual representation highlights five key stages in the development of our understanding of parenting styles: (1) Baumrind's Traditional Framework, (2) Limitations of Baumrind's Model, (3) Alternative Parenting Approaches, (4) Cultural & Contextual Influences, and (5) Emerging Parenting Patterns. Each stage builds upon the previous one, illustrating how contemporary research has moved beyond Baumrind’s original framework to address its limitations and incorporate new insights.

First, Baumrind's Traditional Framework. Baumrind’s model, which categorizes parenting styles into authoritative, authoritarian, permissive, and neglectful, serves as the foundational framework for understanding parenting styles. This model is based on two dimensions—responsiveness and demandingness—and has been widely influential in developmental psychology. However, as the figure indicates, this framework has inherent limitations, particularly when applied across diverse cultural contexts. The second, Limitations of Baumrind's Model, identifies critical shortcomings of Baumrind’s traditional framework. Key limitations include the neglect of emotion coaching and the assumption of cross-cultural validity. These limitations arise from the model’s Eurocentric origins and categorical rigidity, which fail to account for the diversity of parenting practices observed globally. For instance, the figure notes that Baumrind’s model may not adequately capture parenting dynamics in collectivistic cultures or those with different value systems.

Then, the third, Alternative Parenting Approaches. In response to these limitations, researchers have explored alternative parenting approaches that emphasize emotional support, cultural sensitivity, and process-oriented strategies. The figure highlights the importance of emotion coaching and the need for parenting models that are adaptable across different cultural settings. These alternative approaches reflect a shift toward more dynamic and ecologically sensitive frameworks, underscoring the inadequacy of rigid typologies. The fourth, Cultural & Contextual Considerations, emphasizes the role of cultural and contextual factors in shaping parenting practices. As depicted in the figure, cultural biases, contextual relevance, and evolving societal norms significantly influence how parenting styles are conceptualized and practiced. This stage underscores the need for culturally informed models that recognize the interplay between parental behaviors and sociocultural environments. For example, the figure suggests that parenting styles must be understood within the specific cultural context, such as Javanese values of respect for hierarchy (*urutan*) and emotional restraint (*nrimo, alus*).

Next, Emerging Parenting Patterns introduces new, empirically identified parenting patterns that do not fit neatly within Baumrind’s traditional framework. Advanced statistical techniques, such as latent profile analysis, have revealed distinct profiles such as controlling-indulgent, absent, and ambiguous parenting. These emergent patterns highlight the complexity and fluidity of real-world parenting, which cannot be captured by static categories. The figure also points to the rise of hybrid models, such as gentle parenting and versatile parenting, which integrate global ideals with local cultural values.

By synthesizing these stages, the figure illustrates a clear trajectory in parenting styles research: from the establishment of a universal framework to the recognition of its limitations, followed by the exploration of alternative approaches, the incorporation of cultural and contextual influences, and the identification of new, emerging patterns. This progression reflects a growing consensus in the field that parenting should be reconceptualized as a dynamic, bidirectional process shaped by cultural meaning, situational demands, and developmental reciprocity. Ultimately, the figure supports the argument that future research must move beyond rigid typologies toward more inclusive, adaptive, and culturally attuned models to better understand and promote child well-being in diverse cultural landscapes.

Furthermore, advanced statistical techniques such as latent profile analysis have uncovered empirically distinct parenting patterns—such as controlling-indulgent, absent, and ambiguous parenting—that do not fit within the traditional four-quadrant model, underscoring the inadequacy of dichotomous classifications based on arbitrary cut-offs (Kassis et al., 2025; Zheng et al., 2022). These emergent profiles reflect the hybrid, fluid, and often contradictory nature of real-world parenting, which cannot be reduced to static categories. The rise of alternative frameworks—such as Self-Determination Theory-based models Abidin et al. (2022), emotion coaching Gottman et al. (1996), and gentle parenting Pezalla and Davidson (2024)—further illustrates a scholarly and practical pivot toward process-oriented, need-supportive, and relationally attuned approaches that prioritize autonomy, emotional co-regulation, and mutual respect over hierarchical control.

Moreover, this systematic review has critically examined the limitations of Baumrind’s typology and explored culturally adaptive alternatives. While we do not propose a new taxonomy or universal model, our synthesis reveals a coherent shift in how parenting is understood—a reconceptualization grounded in three interrelated paradigmatic movements. First, from Typology to Dimensional Continuum. Baumrind’s model relies on discrete categories derived from dichotomous cut-offs on responsiveness and demandingness. However, contemporary research—particularly latent profile analysis (LPA)—demonstrates that parenting exists along fluid spectra rather than fixed types (Kassis et al., 2025; Zheng et al., 2022). Styles such as controlling-indulgent or ambiguous parenting emerge precisely because real-world caregiving defies binary classification. This shift toward dimensional, person-centered approaches acknowledges variability within individuals and across contexts, moving beyond rigid labels to embrace complexity.

Second, from Universalism to Cultural Relationality. The assumption that responsiveness and demandingness are interpreted uniformly across cultures is increasingly untenable. Instead, the meaning of parental behaviors is mediated by cultural scripts. For example, control may be perceived as coercive in individualistic societies but as protective guidance in collectivistic ones (Agbaria & Mahamid, 2023; Ren et al., 2024). In Javanese culture, values such as *rukun* (harmony), *nrimo* (acceptance), and *tut wuri handayani* (guiding from behind) reflect a relational ethics where authority is exercised indirectly and warmth is expressed through stability, not verbal affirmation (Pohan et al., 2025; Salehuddin et al., 2025). This calls for a cultural relationality lens—one that views parenting not as a set of isolated behaviors, but as embedded in shared meanings and communal expectations.

Third, from Static Labels to Dynamic Practice. Parenting is often treated as a stable trait, yet evidence shows it is highly responsive to developmental stages, situational demands, and structural pressures (Marceau, 2023; Ram et al., 2014). A parent may use authoritative strategies at home but authoritarian tactics in unsafe neighborhoods—a flexibility invisible in typological models. Contemporary frameworks like emotion coaching (Gottman et al., 1996), gentle parenting (Pezalla & Davidson, 2024), and Self-Determination Theory-based models (Abidin et al., 2022) reflect this shift by emphasizing process over category, co-regulation over control, and adaptability over consistency. Together, these three shifts—from typology to continuum, from universalism to relationality, and from static to dynamic—constitute a coherent reconceptualization of parenting research. They do not form a new model to replace Baumrind, but rather a paradigmatic framework that integrates insights from cross-cultural studies, advanced methodologies, and alternative theories into a more inclusive, ecologically valid understanding of effective caregiving.

Moreover, the discussion on parenting styles and their cultural relativity is particularly relevant when applied to Javanese culture, one of the largest and most influential ethnic groups in Indonesia, where collectivism, social harmony (*rukun*), respect for hierarchy (*urutan*), and emotional restraint (*nrimo, ngempet ati*) are deeply embedded cultural values (Kurniati et al., 2017; Pasteruk, 2020). In this context, the universal validity of Baumrind’s authoritative parenting ideal—emphasizing open dialogue, autonomy support, and bidirectional communication—is called into question, as Javanese parenting often blends high control with warmth in ways that align more closely with what Western frameworks might label "authoritarian," yet function adaptively within their sociocultural ecosystem.

In Javanese families, parental authority is traditionally non-negotiable, with an emphasis on obedience (*tut wuri handayani*—a Javanese philosophical concept meaning "guiding from behind," often interpreted as leading through quiet authority rather than permissiveness). Parents are expected to guide, protect, and maintain social order, while children are socialized early to internalize discipline, respect (*ngurmati)* and emotional self-regulation (*kendel, angger-angger rasa*). This aligns with findings by Arafat et al. (2020) and Herzog et al. (2015) that authoritarian practices can be normative and beneficial in collectivistic societies, where such control is perceived not as punitive but as a sign of care, responsibility, and moral instruction. Thus, a parenting style that combines emotional warmth with firm behavioral expectations—what Zheng et al. (2022) identify as controlling-indulgent—may be a more accurate descriptor of effective parenting in Javanese contexts than the Western dichotomy of "authoritative vs. authoritarian."

In addition, Javanese parenting is deeply contextual and relational, emphasizing situational appropriateness (*wewenang, nandheske lan nyesuekke kahanan*) and indirect communication (*nyindir alus utawa nganggo umpomo*) (Kalfin et al., 2024), which resonates with (Darling & Steinberg, 1993) argument that parenting must be understood as a contextual process. The rigid categorization of parenting styles fails to capture the fluidity with which Javanese parents shift between firmness and nurturance depending on the child’s age, gender, social setting, and developmental stage—a nuance highlighted in Khosla and Sharma (2024) and Parameswaran (2021). For instance, younger children may be indulged emotionally (*diwedeni*), while adolescents are expected to demonstrate maturity and restraint, reflecting a dynamic rather than fixed parenting approach.

Furthermore, the emerging concept of gentle parenting (Pezalla & Davidson, 2024), while recently gaining scholarly traction in Western and global discourse, emphasizes empathy, co-regulation, and non-hierarchical dialogue. This framework may appear incongruent with traditional Javanese values that uphold intergenerational hierarchy and emotional modesty. However, modern, urban Javanese families are increasingly adopting hybrid models that integrate global parenting ideals with local values—what Alonso-Stuyck (2019) calls versatile parenting.

Finally, the identification of absent or ambiguous parenting patterns (Kassis et al., 2025) also holds relevance in contemporary Javanese society, where economic migration (e.g., parental work as TKI/TKW abroad) leads to prolonged separation, creating emotionally present but physically absent caregivers (Puspitasari et al., 2021). These complex realities underscore the need for culturally grounded frameworks—akin to the Russian models noted by Shvedovskaya and Archakova (2015)—that move beyond Western typologies to capture the moral, spiritual, and communal dimensions of Javanese parenting, where the goal is not individual autonomy but social harmony, moral integrity (*bathin*), and familial dignity (*martabat keluarga*).

In sum, applying Baumrind’s model uncritically to Javanese culture risks mislabeling adaptive, culturally coherent practices as suboptimal. Future research must develop or validate context-sensitive instruments—such as culturally adapted versions of the ESPA29 (Martínez et al., 2017) or the Parenting Behaviours and Dimensions Questionnaire (Reid et al., 2015)—that reflect the unique interplay of authority, warmth, and relational ethics in Javanese families. Only then can parenting science truly be inclusive, valid, and actionable across diverse cultural landscapes.

### Limitations

This systematic literature review has several limitations that warrant acknowledgment. First, the study was limited by its reliance on secondary data synthesis rather than primary empirical analysis. As such, it cannot establish causal relationships or quantify the relative impact of specific parenting styles across cultures. The conclusions are based on the interpretation and integration of existing studies, which vary in design, quality, and theoretical orientation.

Second, a notable limitation is the restriction to English-language publications. While this facilitated consistent thematic analysis across a large body of literature, it may introduce linguistic and cultural bias by excluding regionally significant research published in other languages—such as Bahasa Indonesia, Portuguese, Arabic, Mandarin, or Spanish. For instance, foundational work on Javanese cultural values like *rukun*, *nrimo*, and *tut wuri handayani* may be more deeply explored in local academic sources that were not accessible within this language scope. Future reviews should consider incorporating multilingual databases and translating key non-English works to ensure a more globally inclusive representation of parenting scholarship.

Third, the temporal scope of the review—from 1966 to 2025—spans nearly six decades during which research methodologies, theoretical constructs, and measurement tools have evolved significantly. Older studies often employed observational methods with smaller samples, while recent research increasingly uses longitudinal designs, latent profile analysis (LPA), and culturally validated instruments. To address this heterogeneity, our synthesis prioritized higher-quality, peer-reviewed studies and gave greater weight to contemporary findings when identifying emerging patterns. Nevertheless, we acknowledge that methodological differences over time may affect the comparability of results, even as the persistence of certain themes—such as the contextual validity of authoritarian parenting—across eras strengthens their credibility.

Fourth, while this review highlights broad cultural patterns, it risks oversimplification by treating cultural groups—such as "Javanese society"—as relatively homogeneous. In reality, significant intra-cultural variations exist based on socioeconomic status, urban versus rural residence, religious affiliation, generational change, and exposure to globalization. Parenting in urban Yogyakarta may differ markedly from rural Central Java, and families may blend traditional values with modern, Western-influenced practices—a phenomenon reflected in the rise of hybrid models like versatile parenting (Alonso-Stuyck, 2019). Future research must move beyond aggregate cultural labels toward more granular analyses that capture internal diversity and dynamic identity negotiation.

Finally, the qualitative, thematic nature of this synthesis, while appropriate for exploring complex, context-sensitive phenomena, lacks the statistical power of a meta-analysis. A quantitative approach could provide effect sizes and comparative strength of associations across contexts. However, given the high degree of conceptual and methodological heterogeneity among the included studies—including diverse definitions of parenting dimensions and outcomes—a meta-analysis would risk ecological fallacy or inappropriate generalization. Instead, our choice of thematic synthesis aligns with best practices for interpretive, theory-building reviews (Sandelowski & Barroso, 2007); Thomas & Harden, 2008). We recommend that future studies conduct focused meta-analyses within more homogeneous cultural or methodological subgroups to complement this broader critical synthesis.

## Conclusion

This systematic literature review argues that while Baumrind’s parenting typology has been foundational in developmental psychology, its applicability as a universal model is limited in a culturally diverse, globalized world. Rooted in Western, individualistic values, the traditional categories—authoritative, authoritarian, permissive, and neglectful—fail to account for the cultural specificity and fluidity of parenting practices observed across different societies. Cross-cultural research shows that authoritarian parenting can be adaptive in collectivistic contexts (e.g., African American, Arab, Asian), while indulgent styles yield positive outcomes in Latin cultures, challenging the assumed superiority of authoritative parenting. Moreover, advanced analytical methods and alternative frameworks—such as Self-Determination Theory, emotion coaching, positive discipline, and the ESPA29 model—highlight the importance of autonomy support, emotional attunement, and contextual adaptability, moving beyond rigid dimensions of warmth and control. The Javanese emphasis on harmony (*rukun*), respect (*ngurmati*), and emotional restraint (*nrimo, ngempet ati*) further illustrates how culturally embedded practices defy Western categorizations yet remain effective within their context. Consequently, the review calls for a paradigm shift from static, Eurocentric typologies toward dynamic, culturally responsive models that view parenting as a reciprocal, contextually mediated process, urging future research to adopt interdisciplinary, longitudinal, and mixed-method approaches with culturally sensitive tools to build a more inclusive, equitable, and globally relevant science of parenting.

## Data Availability

The data supporting the findings of this systematic review are derived from the included studies, which are cited in the reference list. All data analyzed during this review are available in publicly accessible academic databases (Scopus, Web of Science, PubMed, and Google Scholar). The supplementary material, including the full list of included studies and thematic synthesis table, can be made available from the corresponding author upon reasonable request.
